# The status of refrigeration solutions for last mile vaccine delivery in low-income settings

**DOI:** 10.1016/j.jvacx.2022.100184

**Published:** 2022-06-18

**Authors:** Magali Cattin, Sashidhar Jonnalagedda, Solomzi Makohliso, Klaus Schönenberger

**Affiliations:** EssentialTech Centre, Ecole Polytechnique Fédérale de Lausanne, Station 10, 1015 Lausanne, Switzerland

**Keywords:** Vaccine transportation, Cold chain, Off-grid refrigeration, Last mile delivery

## Abstract

Recommendations for storage of most vaccines imply a continuous exposure to a temperature range between 0 °C and 10 °C, from the production to the administration to beneficiaries. According to the World Health Organization, more than 50% of vaccines are wasted around the world. Discontinuities of the cold chain in low-income settings where electricity is scarce contributes to this wastage. Recently, several advances have been made in cooling technologies to store and transport vaccines. This paper presents an overview of refrigeration technologies based on scientific publications, industry white papers and other grey literature. With a focus on vaccine transport, we briefly describe each refrigeration method, its best performing available devices as well as the outstanding research challenges in order to further improve its performance.

## Introduction

**Fig. 1:**
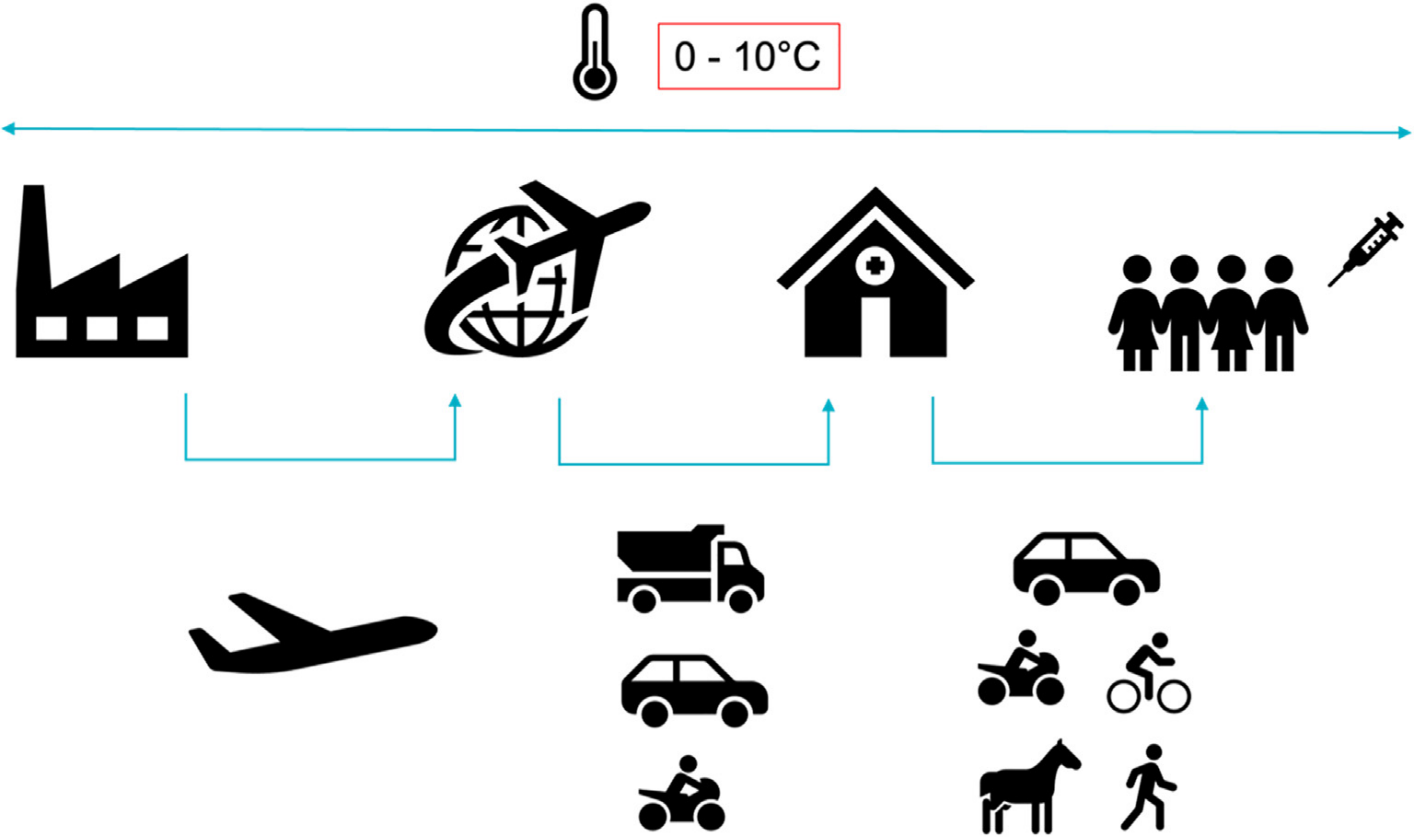
**Cold Chain Process**, from manufacturing (on the left) to beneficiaries (on the right) with a requirement for temperature between 0 and 10 °C during the whole chain and various means of transport according to the stage of the cold chain.

Each year, more than 1.5 million people die from a disease that could have been prevented with vaccination [1]. In order to maintain their full potency, all vaccines require to be stored at specific temperature ranges to be complied with, from manufacturing to beneficiaries. While this temperature requirement varies according to the type of vaccine and the stage of the supply chain, or cold chain (illustrated in Fig. 1), most of essential vaccines such as against measles, tuberculosis, diphtheria, tetanus, pertussis or hepatitis need to be stored between 0 °C and 10 °C through the whole cold chain [2].

According to the World Health Organization (WHO) (2005), more than 50% of vaccines are reported to be wasted around the world [3]. In a more recent study in South Sudan, the wastage of tetanus-diphtheria vaccines even goes up to 57% [4]. In addition to vaccines being discarded or unused, one protruding reason for this wastage is the inability to maintain the optimal temperature range along the cold chain, vaccines being either exposed to risks of freezing or to heat [5]. Apart from the waste of resources, this situation can limit the access to essential vaccination and induce potency loss of the vaccines due to exposure to temperatures outside the appropriate range. The consequences can be fatal for the compromised individual and favour the spread of viruses that are on the list for eradication.

According to the amount of vaccines to be carried, each stage of the cold chain (national, regional, villages) requires specific transport and storage equipment [6]. While the choice of transport mode is mainly influenced by road conditions, the choice of storage equipment depends on the availability of energy resources (mainly electricity), the duration of transportation or storage as well as the volume of vaccines [7]. The vaccine cold chain involves accessibility and transportation challenges [8], particularly for last mile delivery in low-income settings where appropriate equipment might be lacking, electrical supply can be erratic - if available at all - roads can be impassable and health workers may not be adequately trained [9].

To ensure a safe and effective delivery of immunization, the WHO has developed the Performance, Quality and Safety (PQS) system [10]. This provides cold storage recommendations specific to each stage of the supply chain and reports the performance specifications and test procedures to prequalify cold chain equipment. To help procurement agencies make decisions, the PQS catalogue [2] lists prequalified cold chain equipment. The refrigeration equipment falls into two main categories: passive and active [11].

Passive refrigeration devices do not require any external source of energy during use and are thus considered as off-grid solutions. Such equipment consists of (i) long-term passive containers, which are used to store vaccines at health facilities where electricity is unreliable, (ii) cold boxes, typically used for distribution of vaccines between facilities and (iii) vaccine carriers, used to transport vaccines from the facilities to the beneficiaries, for outreach and immunization sessions. The performance of passive refrigeration equipment is mostly assessed by its “cold life”, i.e., the duration it is able to maintain an inner temperature range of 0–10 °C.

Active refrigeration equipment requires access to energy in the form of heat or electricity during use. This approach is more appropriate for storage in health facilities than for transportation. When electricity is available 8 h or more per day and power outages are shorter than 48 h, on-grid equipment is recommended by the WHO. On the contrary, when electricity is available less than 8 h per day with recurrent power outages longer than 48 h, devices able to generate their own power such as heat-driven or solar refrigerators are preferred [7]. Historically environmentally harmful refrigerants were used for refrigeration purposes. More recently, refrigerants with low ozone depletion potential (ODP) and low global warming potential (GWP) have been developed, relying on natural substances [12], [13].

The performance of an active refrigeration system is represented by its efficiency to convert input energy such as electricity or heat to cooling capacity (i.e. capacity to remove heat from a cold source). This measure is called the coefficient of performance (COP) and is computed as the ratio between cooling capacity (output) and supplied energy (input).

Especially under current circumstances of the Covid-19 pandemic, it is very important to review existing solutions and propose new directions of research. The scope of this article includes highlighting the main refrigeration methods for last mile vaccine delivery applications, discussing the technology status, comparing their performance if already available, and underscoring the remaining technological challenges to be addressed. The article is organized as follows, it first introduces the three major cooling methods: Insulated box and coolant packs-based systems, Sorption refrigeration and Thermoelectric refrigeration. In each of these subsections, the working principle of the respective method, its performance (efficiency and/or cold life) and the devices currently in development or already available on the market are discussed. Section “Discussion” then reviews the key findings, compares the different refrigeration methods and points out the limitations of the study as well as future perspectives. Section “Conclusion” places this research in the perspective of its context and constraints.

## Refrigeration solutions for vaccine transport

An ideal vaccine carrier or cold box, essential for last mile delivery, should show high efficiency in ensuring a storage temperature between 0 and 10 °C for a long duration (min. 15 h according to the WHO PQS), while maximizing vaccine storage volume. It should be portable (i.e. light) and robust with minimal recharging constraints.

Table 1 provides a summary of the refrigeration solutions for vaccine storage and transport, their corresponding state of development, advantages, disadvantages and research challenges. These are described in more detail below.

**Table 1:** **Overall assessment** of the various refrigeration techniques for vaccine applications. Note about COP ranges: for absorption systems, it corresponds to NH3-LiNO3 absorption cooling systems for small scale applications; for adsorption systems, residential scale application and solar-powered systems; for thermoelectric systems, space cooling and food refrigeration applications.

Refrigeration method	State of Development		COP	Advantages	Disadvantages	Research challenges
	Prototype	Market				
**Insulated box and coolant packs**	–	Cold Box, Vaccine Carrier	*NA*	- Low cost - Low maintenance	- Coolant packs: Preparation and conditioning, risk of freezing, additional weight - Low volume ratio vaccines/coolant packs	- Insulation materials with low thermal conductivity - PCM with low density and low thermal conductivity
**Absorption**	Vaccine Carrier	Refrigerator	0.05–0.74	- Low-grade heat (75–120 °C) - Few moving - Low ODP and GWP refrigerants	- High initial costs - Low COP - Bulky	- Cost reduction - Increase of system efficiency
**Adsorption**	Cold Box	Industrial application	0.078–0.81	- Low-grade heat (50–100 °C) - Very few moving parts - Robust to vibration - Low ODP and GWP refrigerants	- No technical maturity - Low COP	- Heat and mass transfer efficient working pairs
**Thermoelectric**	–	–	0.1–0.15	- Compactness, light weight - No moving part - Environmentally friendly	- Low COP - Use of batteries	- Low-cost material with high figure of merit ZT

### Insulated box and coolant packs-based systems

#### Working principle

The most common passive refrigeration systems rely on the combination of an insulated container with coolant packs acting as thermal batteries to control vaccine temperature. A container is efficiently thermally insulated when heat exchange between its interior and exterior is minimized. While most solutions rely on the use of insulative foam, one strategy to achieve this consists in placing two flasks one within the other, joined at the neck and creating a near-vacuum gap between the two recipients, to reduce heat transfer [14]. This principle of vacuum flasks is also applied in cryogenic dewars for which two flasks are separated with vacuum and multilayer insulation [15]. However, both these systems present some drawbacks: creating the near-vacuum is highly energy-demanding, maintaining this state requires expensive materials, and leakages are difficult to detect and identify. As a consequence, the temperature inside the container can increase and affect the vaccine's potency, without the knowledge of the user. [16].

The thermal batteries placed inside the container to control its temperature consist of packs filled with phase change material (PCM). PCM is a substance that releases or absorbs sufficient energy during phase transition (e.g. from solid to liquid, or from liquid to gas) to generate either a heating or cooling effect on the surroundings [17]. When the PCM condenses or freezes, it releases a large amount of energy in the form of latent heat. When the PCM melts or evaporates, it absorbs energy from the environment. The coolant packs need to achieve a temperature range of 0–10 °C [18] and thus when the packs are frozen, pre-conditioning of coolant packs is recommended (i.e. exposure to ambient temperature until liquid water appears) before use in order to avoid freezing of the vaccines [19]. The WHO recommends water as a simple, cheap, safe and effective PCM to use, despite its freezing temperature of 0 °C necessitating pre-conditioning to avoid damaging the vaccines. The WHO PQS states that the only PCM packs allowed to be removed by the user are filled with water.

The WHO PQS system divides the passive refrigeration technologies into various categories depending on their vaccine storage capacity and adjusts its cold life requirements accordingly (see Table 2) [20]. The main difference between vaccine carriers and cold boxes is their storage capacity, 0.5–5 L for vaccine carriers (up to 50 vials), up to 100 L for large storage capacity cold boxes (up to 1,000 vials). Some of those technologies are freeze-preventive due to an additional freeze protective layer. For last mile delivery conducted by a single health worker, vaccine carriers are the most relevant option: such system is required to have a cold life of a minimum of 15 h and is considered as being long range if above 30 h.

**Table 2:** **Cold holdover requirements** for vaccine carriers and cold boxes, based on Specifications from WHO PQS Catalogue [20].

	Capacity	Short range	Long range
Vaccine carrier	0.5–5 L	>15 h	>30 h
Long-term cold box	5 L	–	>35 days
Conventional cold box	5–25 L	>48 h	>96 h
Large storage capacity cold box	>100 L	>24 h	>48 h

Each figure below is presented with their respective title and caption. All figures can be represented in grey levels for printed versions.

While passive refrigeration is appreciated for its low cost, low maintenance and light weight, it presents some limitations [19]. Firstly, freezing and preparing the coolant packs requires access to active refrigeration, which involves adequate electrical power. Additionally, passive containers for vaccine transport generally offer limited storage time and are thus more appropriate for single day missions, or when substations are available, where coolant packs can be recharged and replaced by newly conditioned packs. Finally, the use of such passive refrigeration poses a risk that the vaccine could freeze due to incorrect use of the coolant packs or use of the wrong type of container.

#### Existing technologies

Even though vaccine carriers and cold boxes are already widespread on the market, the use of coolant packs is constraining and their cold life could still be improved. So far, only one container ensuring a long-term storage (>35 days) has been prequalified by the WHO PQS programme, namely the Arktek Passive Vaccine Storage Device (PSD).

Developed in collaboration with the Bill & Melinda Gates Foundation, the Arktek PSD provides a 5.4 L vaccine storage capacity [21]. It relies on the principle of a vacuum flask with multilayer insulating materials (i.e. cryogenic dewar) and is not powered by electricity but requires ice packs to be renewed monthly. When the ambient temperature goes up to 43 °C, the device ensures a temperature in the range of 0–10 °C for at least 35 days [22]. The Arktek PSD is currently sold to leading healthcare stakeholders (WHO, Médecins Sans Frontières, UNICEF, etc.), despite its non-PQS prequalification for transport.

Another type of long-term cold box, based on Vacuum Insulated Panels and providing freeze protection, was developed by the Sure Chill company, also with the support of the Bill & Melinda Gates Foundation [23]. The device with a vaccine storage capacity of 7.8 L was field tested and able to maintain an internal temperature below 10 °C for 33–42. It is undergoing further field trials. However, no evidence was found of WHO prequalification status.

#### Research challenges

In passive devices, due to the thickness of the insulation and the number of coolant packs, the effective volume of vaccines transported in the container is as low as 8% compared to its total volume [2]. The main research efforts thus aim to improve the performance of passive containers and increase their storage capacity by focusing on heat retention. The objective is to develop a low-cost and high-performance insulation, which minimizes heat leakage while reducing the volume of the coolant packs.

*Thermal insulation* | Super Insulated Materials, such as Vacuum Insulated Panels and aerogels, offer an alternative approach for thermal insulation [24].

• Vacuum Insulated Panels (VIPs) ensure a low thermal conductivity through their composition and structure, i.e. vacuum trapped in a polymer matrix comprising a core and an envelope. VIPs performance decreases over time due to the permeation of gases or water vapour through the envelope [25]. Moreover, such material is fragile as the insulation performance would decrease significantly if punctured. This could be overcome with the development of durable materials that better resist permeation but application in low-income settings would require investigation on their cost [26]. While VIPs are common for application in the field of building and construction, they have more recently been used for transport of vaccines, for instance by the company Va-Q-Tec [27]. Further research is required to assess their applicability for storage of vaccines in arid and tropical climates, in particular as regards the potential influence of environmental moisture on their efficiency.
• Aerogels consist of nanoporous materials with pore sizes around 20 nm and variable mass density [28]. They are solids derived from gel where the liquid component is replaced with gas and could for instance be used as a core material for VIPs. Aerogels show non-flammability, non-reactivity, low thermal conductivity at ambient pressure and adjustability in terms of size and shape. Despite these valuable properties, their cost of production remains high, they are humidity-sensitive and show a low tensile strength, making the materials fragile [26].

*Phase Change Materials* | When designed with a freezing temperature in the range required for vaccine storage (e.g. 5 °C), PCMs have the potential to reduce the risk of freezing of coolant packs by creating a thermal buffer zone. Such alternatives to water PCM could be organic (e.g. paraffin, fatty acids or vegetable oils), inorganic (e.g. salt hydrates or metals) or eutectic types [17]. Most of these PCMs come with issues that are not encountered when using water, such as environmental concerns about their disposal due to their non-biodegradability, potential toxicity (i.e. skin and eye irritation), or incompatibility with the container. However, material exchange with the environment (leakages) can be prevented, through encapsulation, by providing a support structure to the PCM [29]. The encapsulation can be either macro-, micro- or nanoscale. While macro-encapsulation consists of placing PCM in pouches or other containers, micro-encapsulation generates PCM droplets coated with another material. Macro-encapsulation improves the compatibility of PCM with surrounding material and improves PCM handling during production. However, the PCM heat transfer characteristics can be altered, resulting in lower performance. Micro-encapsulation reduces the risk of leakage while increasing the surface area to improve heat transfer, and thus potentially reducing the volume of PCM required compared to vaccine volume. More recently, based on the same principle than micro-encapsulation but at smaller scale, nano-encapsulation is also under investigation [30].

### Sorption refrigeration: absorption and adsorption

#### Working principle

Sorption refrigeration systems function in a similar as classic compression refrigerators [31]. However, instead of using electricity to function, they use low-grade heat (low temperature). Absorption and adsorption cooling methods rely on this approach. While the absorbate is dissolved into the absorbent in the process of absorption, the adsorbate adheres to a surface of the adsorbent in the process of adsorption [32]. The coupling of an absorbate/adsorbate and an absorbent/adsorbent is called a working pair.

**Fig. 2:**
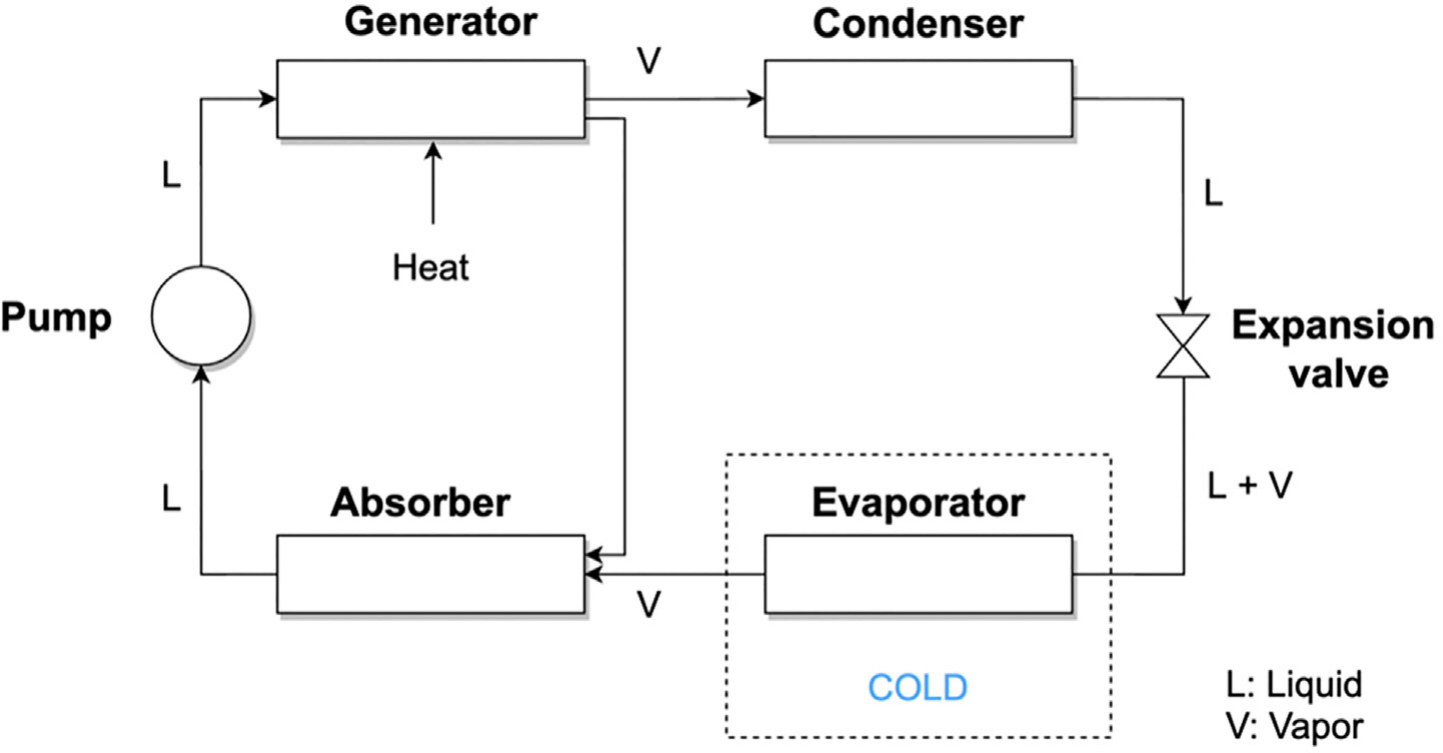
**Single absorption process**[32]. Heat – typically between 75 °C and 120 °C [31] – is applied to the generator in which a refrigerant-saturated liquid is located. Upon heating, the refrigerant becomes a high-pressure and high-temperature gas that liquefies when it is injected into the condenser. The refrigerant is then depressurized through the expansion valve, producing the cooling effect. The evaporator then absorbs latent heat of vaporization from its environment. The gaseous refrigerant is finally absorbed again in the absorber and the cycle starts again.

*Absorption |* The working fluid of an absorption refrigeration system consists of a liquid mixture of absorbate and absorbent [33]. In addition, the system consists of a generator, a condenser, an expansion valve, an evaporator, an absorber and a pump, driven by external heat (see Fig. 2). The most common working pairs used for absorption refrigeration consists of water combined with salts, acids or bases in solutions such as lithium bromide (LiBr), or with ammonia (NH_3_).

Absorption coolers use refrigerants with low ODP and low GWP, require a low-grade heat (75–120 °C) and are more technically mature than adsorption cooling systems [34]. However, they have a high upfront cost, are usually bulky and achieve low efficiency so far, e.g. COP values ranging between 0.05 and 0.74 for NH_3_-LiNO_3_ absorption cooling systems for small scale applications [35].

**Fig. 3:**
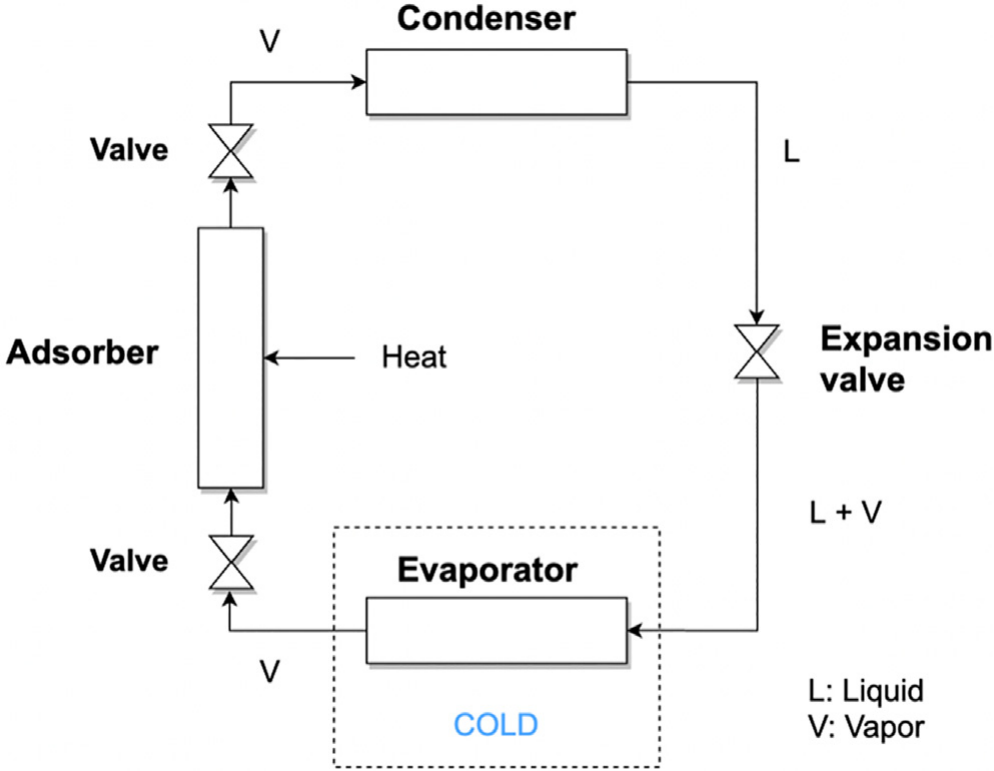
**Single adsorption process**[35]. At the beginning of the cycle, heat – typically between 50 and 100 °C [31] – is applied to the adsorbent bed (adsorber) saturated with refrigerant/adsorbate: the pressure of the adsorbate increases and the refrigerant is vaporized. The vapour then flows to the condenser where it undergoes phase transition to liquid. The liquid refrigerant flows to the expansion valve, where its pressure and temperature are decreased, thus providing the overall cooling effect. The refrigerant finally goes through the evaporator and absorbs latent heat of vaporization from its environment. which is the main mechanism providing. The cycle can start again after the refrigerant has been re-adsorbed by the adsorbent bed.

*Adsorption* | Adsorption refrigeration operates very similarly to absorption systems. They consist of an adsorber, a condenser, an expansion valve and an evaporator (see Fig. 3) [36]. The adsorbent is a porous solid element which should exhibit a high capacity for adsorption with temperature variation, such as when exposed to heat [37]. The adsorption capacity of a working pair is assessed as the maximal amount of adsorbate (grammes) that can be taken up by the adsorbent (grammes), i.e. g_adsorbate_/g_adsorbent_: the higher the amount of adsorbate that can be adsorbed by the adsorbent, the longer the refrigeration period, since the adsorbent takes in more refrigerant fluid. While common adsorbents consist of activated carbon, silica-gel and zeolites, common adsorbates consist of ammonia, methanol and water.

Adsorption coolers also use refrigerants with low ODP and GWP, require even lower grade heat (50–100 °C) and are suitable for applications where there is a lot of vibration because of their low number of moving parts, a feature desirable in rough road conditions [38]. However, the technology has not yet reached technical maturity for small devices applications such as vaccine carriers and research needs to be conducted to improve efficiency [35], typical COP values for residential scale application (solar-powered systems) are ranged between 0.078 and 0.81 [39].

Sorption systems could be coupled with solar systems [40], [41], thermal energy storage devices or even waste heat recovery [42], [43] in order to provide higher performance and/or cost reduction. In addition to reducing long-term costs, powering sorption systems with waste heat recovery or renewable energy to drive the desorption phase could also reduce its environmental impact [44].

#### Existing solutions

Commercially available sorption solutions are found in various fields such as air-conditioning, ice making [41], [45] and vaccine storage [46] but not in vaccine transport (carriers). The WHO PQS system encompasses requirements for absorption systems but adsorption solutions are not mentioned. While adsorption still faces significant technical challenges, absorption technology is mature, showed its efficiency in various application and was widely used for vaccine storage before being phased out due to their need of supply in gas or kerosene [47]. Despite the potential of sorption technologies in vaccine storage, none has yet been prequalified by the WHO for safe vaccine delivery even though some devices are under development. A major barrier to overcome relies on the method for recharging of those technologies which requires a significant amount of energy.

*Example of absorption cooling transport solution* | The ISOBAR cooling technology [48] is an absorption-based vaccine box showing 88 h of cooling under WHO PQS test conditions (theoretically even 130 h). It uses ammonia and water as a working pair that can be pressurized or charged electrically or thermally. The device does not seem to be available on the market and the advancement of research could not be found.

*Example of adsorption cooling solution* | In 2019, Enersion Inc. filed a patent [49] for a cold storage adsorption-based container in which the top of an insulated vacuum chamber is covered with an adsorbent material which adsorbs water vapour from melting ice. They compared the performance of cold boxes without adsorbents, with silica gel adsorbent or with zeolite adsorbent: the cold box with silica gel provided a cold hold-over time below 15 °C of 187 h which is 7.6 times greater than the cold box without adsorbent.

A promising adsorption material-based device was developed with the support of the Bill & Melinda Gates Foundation: the Indigo Cooler [50], [51]. Two metallic containers are placed one within the other, the wall of the inner container is filled with low pressure water and the wall of the external insulated container is a near-vacuum area. Both containers are connected by a valve. When opening the valve, the water evaporates from the inner container to the near-vacuum area in the outer container, thus creating a cooling effect in the vaccine compartment. The device can be recharged with a specific charger applying heat to move back the water from the outer container to the inner one. In that way, the same water can thus be used for several cycles of refrigeration.

The Indigo vaccine carrier has a 2 L inner storage capacity and can be worn like a backpack. The device does not require any electricity, ice or battery during use. The pressure inside the device is initially lowered so that water evaporates at 5 °C. The device allows the storage temperature range to be maintained for 5 days when the ambient temperature is 43 °C. No evidence was found of the Indigo Cooler being available on the market or whether it has been PQS-prequalified.

Another example of adsorption cooling solution is developed by the Coolar company in Germany [52]. It is a stationary vaccine storage solution and consists of an adsorption refrigeration system relying on warm water. Due to this latter characteristic (rather than relying on electricity), the company claims their device to be close to a carbon neutral solution.

#### Research challenges

In order to bring drastic improvement to the efficiency of sorption systems, the main research challenge is to develop working pairs that have considerable heat and mass transfer properties [36].

*Absorption |* On most widely used and efficient working pairs – NH3/H2O and H2O/LiBr – are based on volatile solutions and NH3/H2O even requires a vapour purification process to ensure the quality of the refrigerant [53]. Alternatives, showing potential even though requiring further investigations, have emerged, with ternary and quaternary salt mixtures as refrigerants [33]. For instance, LiNO3 or NaSCN show potential in replacing water in the ammonia (NH3)/water (H2O) pair.

*Adsorption |* The research focus is on adsorbents: composite adsorbents to enhance refrigerant uptake, creating an adsorbent coating over the heat transfer metal surfaces to decrease thermal resistance, or using Metal-Organic Frameworks (MOFs) [36]. MOF materials consist of highly porous materials offering robust and stable structures with tunable characteristics regarding their architecture and functionalization. Rezk *et al.* [54] identified one specific MOF, HKUST-1, to increase the water uptake by 93.2% compared to silica gel performance. Commercially available refrigerants used for other refrigeration systems could also be employed as adsorbate to achieve a higher adsorption capacity [55]. However, even if this reduces problems related to material compatibility and leakages, such refrigerants have environmental drawbacks. A trade-off needs to be found between system efficiency, costs and environmental impact.

### Thermoelectric refrigeration

#### Working principle

The thermoelectric effect relies on heat transfer due to direct current going through an electrical junction [56]. Electrical junctions are made of two materials, conductors or semiconductors, and could be of various types. The p-n type junctions, made of semiconductors, exhibit a very strong thermoelectric effect. The performance and efficiency of a thermoelectric material is evaluated using a dimensionless quantity (ZT): the higher the value, the higher the efficiency [57]. The best thermoelectric materials have a high electrical conductivity and low thermal conductivity (i.e. high thermal insulation) and induce an electrical voltage in response to temperature difference across a material.

The advantages of a thermoelectric cooling system are its compactness, light weight, low noise, proportional control capability, as well as the absence of moving parts and harmful refrigerants [58]. However, thermoelectric systems rely on heavy batteries which require regular replacement and maintenance. While the efficiency of these devices on the market remains very low (COP = 0.1–0.15) due to the lack of appropriate and low-cost materials [59], the performance is even worse when the ambient temperature is high, such as in LMICs [57].

#### Existing technologies

While thermoelectric devices are mostly available in the market for niche applications, several prototypes have been built and studied for vaccine storage or transport. For instance, Gastelo-Raque *et al.* [60] developed a thermoelectric refrigerator system combined with batteries and photovoltaic panels for rural areas. The device stores up to 5 L of vaccines between 4 °C and 6 °C with an autonomy of 72 h based on a rechargeable battery. More recently, Reid. *et al.* [61] presented a proof-of-concept for a thermoelectric chip combined with an aluminium block for storage of the vaccine vials. In contrast to most other designs tested at room temperature, this one is assessed with an ambient temperature of 37 °C and provides an autonomy of 10 h with a single battery charge.

Alternatively, the *Arktek Solar Direct Drive* is an enhanced version of the *Arktek PSD* (see Section “Insulated box and coolant packs-based systems”), including a thermoelectric module. Li *et al.* [62] replaced two of the 8 ice blocks required by the *Arktek PSD* with an active Peltier-based cooling system, connected to a small solar panel. Ensuring a temperature between 3 and 5 °C in the main compartment, a holdover time of 8 days was measured for this system in a controlled environment of 43 °C during the day and 25 °C during the night-time. However, this result is poor compared to the 35 days of the *Arktek PSD*, as a passive refrigerator with 8 packs and no thermoelectric module. This set up also allows to refreeze warm ice packs.

Currently, no thermoelectric device has been prequalified by the WHO. In addition to their low autonomy and efficiency compared to vapour compression alternatives and passive systems, the development of low-cost materials with high figure of merit (ZT) remains a significant technical barrier.

#### Research challenges

Most of the current research regarding thermoelectric refrigeration focuses on improving the ZT value of the materials while reducing their costs. Bismuth telluride (Bi2Te3) based alloys (ZT≈1.0) are the most used for applications around room temperature [58]. Zolriasatein *et al.* [63] showed that the grinding process of a material could influence its thermoelectric properties: as they added stearic acid during the grinding, the figure of merit of the material was enhanced by 15%. Hinterleitner *et al.* [64] developed a new material, made from a thin layer of iron, vanadium, tungsten and aluminium applied to a silicon crystal, which ensures a ZT value between 5 and 6 at 300 °C. However, these materials are very far from commercial application and ZT measurements are very complex and difficult to replicate [65].

## Discussion

### Key findings

For last mile delivery and small capacity applications, cold boxes and vaccine carriers with insulative foam are employed, combined with coolant packs. However, the main limitation for such devices is their use of a large proportion of coolant packs to keep cool a low quantity of vaccines. In addition, coolant packs also require access to a fridge or freezer to reach the appropriate temperature range, incurs constraint of conditioning and a possible risk of freezing. For the freeze preventive solutions, the vaccine storage capacity is reduced by the protection layer. In terms of state-of-the-art insulation, creating near-vacuum space is highly energy-demanding and leakages are difficult to identify. Although super-insulated materials such as VIPs and aerogels are an alternative to near-vacuum by providing a structure and composition with low thermal conductivity, their cost remains a concern for an application in low-income settings.

Promising alternatives to double flasks combined with coolant packs systems include sorption or thermoelectric systems, both of them with benefits, drawbacks and specific challenges. Sorption technologies relying on low ODP and GWP refrigerants are driven by reduced amounts of thermal energy, which could come from waste heat, and require few if any moving parts, which tends to improve their durability. However, their performance is highly influenced by the materials used as working pairs and, for now, absorption refrigeration shows better performance than adsorption due to their technical maturity.

Thermoelectric technologies offer compactness, light weight, low noise, absence of refrigerants or moving parts, but require heavy battery and more efficient materials to reach better performance than currently. Also, they require access to electrical supply which is usually unavailable in the last-mile delivery. With more efficient materials, this technology could be a promising alternative to bulky systems.

Other refrigeration approaches, including magnetic [66], [67] and thermoacoustic cooling [68], [69], present potential for vaccine storage and transport. However, no prototype or device based on these principles has been found for that application area and such systems are currently grid dependent.

### Limitations

This study shows potential limitations. First, this paper is theoretical as it exclusively relies on literature research: scientific publications, industry white papers and other literature. Then, the methodology of literature research was complex due to the broadness of the field. The goal of the study was not only to provide an overview of the various refrigeration technologies for vaccine transport but also to briefly describe the working principle of each approach, to identify their respective devices in development or in the market and research perspectives. In addition to these various levels of research criteria, private companies do not necessarily publish about their research or industrialized products in the usual databases.

The assessment of the various refrigeration methods is mostly qualitative, considering the benefits and drawbacks. Due to the limitation of the available information, the only possible quantitative comparison relies on COP and cold life which varies between the different devices of a same refrigeration method. A comparison through the cost was not possible due to the immaturity of some devices presented.

### Future research

For all of the presented refrigeration methods, sorption and thermoelectric cooling, the research challenges rely in the materials. Sorption needs heat and mass transfer efficient working pairs while thermoelectric cooling necessitates material with high figure of merit. While developing material to improve the performance of the device (e.g. cold life), one needs to consider the cost and durability of such materials for its applicability in low-income settings and possibly find a balance between cost and performance.

## Conclusion

Continuity of the cold chain is essential for maintaining the potency of vaccines. While the current paper focuses on the last mile delivery of vaccines, the requirements and technical challenges vary according to the stage of the vaccine supply chain (national, regional, villages). Disruption of the cold chain generates avoidable wastage of vaccines and innovative refrigeration systems could be part of the solution. This study provides an overview of the various refrigeration approaches including the most widespread solution – insulated boxes combined with coolant packs - and alternative approaches with the potential to improve the outcomes of the cold chain.

Sorption and thermoelectric cooling are likely to reduce the recharging constraints and the risk of freezing related to the use of coolant packs while improving the vaccine storage capacity for a given volume. However, the efficiency of those methods should be improved for application in vaccine transport and most of research pathways for that purpose rely on the development of efficient and low-cost materials,

In these pandemic times, the various COVID-19 vaccines require storage at different temperatures (between 0 °C and 10 °C, at −20 °C or at −70 °C) which enhances the complexity of the vaccine supply chain and emphasizes its indispensability. The current study highlights the urgency and importance to foster the development of low-cost, energy-efficient robust refrigeration systems and state-of-the-art insulation especially for low-income settings.

## Author contributions

MC, SJ, SM and KS participated in the conception and design of the study; MC and SJ drafted the article, SM and KS revised the article for intellectual content and all authors approved the final version to be submitted.

## Declaration of Competing Interest

The authors declare that they have no known competing financial interests or personal relationships that could have appeared to influence the work reported in this paper.
